# Impact of Diabetes on Platelet Function in Acute Ischemic Stroke Patients Taking Dual Antiplatelet Therapy

**DOI:** 10.3389/fneur.2021.712024

**Published:** 2021-11-04

**Authors:** Yinping Guo, Yi Zhang, Jing Zhao, Lingshan Wu, Zhiyuan Yu, Dan He, Hao Huang, Xiang Luo

**Affiliations:** ^1^Department of Neurology, Tongji Hospital, Tongji Medical College, Huazhong University of Science and Technology, Wuhan, China; ^2^Department of Neurology, The First Affiliated Hospital, Sun Yat-sen University, Guangdong, China; ^3^Guangdong Provincial Key Laboratory for Diagnosis and Treatment of Major Neurological Diseases, National Key Clinical Department and Key Discipline of Neurology, Guangdong, China

**Keywords:** diabetes mellitus, dual antiplatelet therapy (DAPT), ischemic stroke, platelet function, thromboelastography (TEG)

## Abstract

**Objectives:** Diabetes mellitus (DM) is a significant risk factor for ischemic stroke and associated with platelet reactivity. We aim to evaluate the effect of DM on platelet function in acute ischemic stroke patients taking dual antiplatelet therapy (DAPT).

**Methods:** We consecutively included patients with acute ischemic stroke taking DAPT. Platelet function was assessed by thromboelastography and the arachidonic acid (AA) or adenosine diphosphate (ADP) induced platelet inhibition rate were used to confirmed the high-residual on-treatment platelet reactivity (HRPR) to aspirin or clopidogrel. We classified patients into DM and non-DM groups. The association between DM and platelet function was assessed and the confounding factors were adjusted by propensity score matching (PSM) analysis. The independent risk factors of HRPR were determined by multivariate logistic regression analysis.

**Results:** A total of 1,071 acute ischemic stroke patients, 712 in the non-DM group and 359 in the DM group, were included. Patients with DM had a significantly higher maximum amplitude (63.0 vs. 62.0 mm, *P* < 0.01), ADP-induced clot strength (34.6 vs. 30.3 mm, *P* < 0.01) and clopidogrel HRPR rate (22.6% vs. 17.3%, *P* = 0.038) than those without DM. Among 662 patients after PSM, the maximum amplitude (63.1 vs. 62.5 mm, *P* = 0.032), ADP-induced clot strength (34.6 vs. 29.3 mm, *P* < 0.01) and clopidogrel HRPR rate (23.0% vs. 15.7%, *P* = 0.018) is still higher in the DM group. DM was an independent factor of clopidogrel HRPR (OR = 1.48, 95% CI: 1.03–2.07, *P* < 0.05).

**Conclusions:** In acute ischemic stroke patients taking DAPT, DM is associated with increased platelet reactivity and higher prevalence of clopidogrel HRPR.

## Introduction

It is well-known that diabetes mellitus (DM) could increase the risk for cerebrovascular diseases. It has been reported that 25–45% of ischemic stroke patients have confirmed DM (1–3). Previous studies have suggested that DM substantially increases the risk for first ischemic stroke (4–6). In addition, DM is associated with increased mortality (7) and recurrence rate of ischemic stroke (8–10).

In the acute treatment and secondary prevention of ischemic stroke, aspirin and clopidogrel are the most widely used antiplatelet drugs in clinical practice. They not only significantly reduce the mortality and disability rate, but also effectively prevent stroke recurrence (11). However, 10–20% of ischemic stroke patients will have new vascular events in the first 3 months despite have received antiplatelet therapy (12–14). One possible reason is the high residual on-treatment platelet reactivity (HRPR), which means the reduced platelet inhibition rate and absence of antiplatelet effect (15).

Studies have found that HRPR may increase the prevalence of ischemic events in coronary heart disease and diabetes patients (16, 17), which may be due to generally higher aggregation activity of platelet and less responsiveness to antiplatelet drugs (18) in patients with DM. Most previous studies on platelet function have focused on individuals with cardiovascular disease. However, studies on the relationship between DM and platelet function in acute ischemic stroke patients taking dual antiplatelet therapy (DAPT) remain scarce. The present study aimed to evaluate whether DM had an effect on platelet function in acute ischemic stroke patients taking DAPT.

## Methods

### Study Population

This study retrospectively included consecutive patients with acute ischemic stroke hospitalized in the Department of Neurology, Tongji Hospital, from September 2013 and May 2019. The inclusion criteria included: (1) ≥18 years old; (2) diagnosis of acute ischemic stroke on the basis of clinical symptoms and magnetic resonance imaging or computer tomography; (3) having received treatment of clopidogrel (75 mg/day) plus aspirin (100 mg/day) without a loading dose for at least 7 days before platelet function testing. Patients would be excluded if: (1) having medications within the past 3 months affecting blood coagulation function, such as cilostazol, warfarin, dabigatran, heparin, and factor Xa inhibitors (such as rivaroxaban); (2) a history of malignant tumors, digestive diseases, or severe liver, kidney, or blood-related diseases.

This study has been approved by the Tongji Hospital Ethics Committee (No. TJ-IRB20210107). The requirement of informed consent was waived since all data analyzed in this study were anonymized and cannot do any harm to the subjects.

### Clinical Assessments

The demographic and clinical information included: age, sex, smoking (defined as a history of smoking ≥1 cigarette per day for 1 year or more), alcohol intake (defined as weekly alcohol intake exceeding 200 g for 1 year or more), a history of ischemic stroke/transient ischemic attack, hypertension, hyperlipidemia, coronary heart disease (defined as a history of myocardial infarction or angina pectoris) and DM (defined as a HbA1c ≥ 6.5%, or a 2 h plasma glucose ≥ 11.1 mmol/L during an oral glucose tolerance test, or self-reported history of DM) (8, 19, 20). Laboratory test were conducted within 24 h of admission, included creatinine, eGFR, platelet indexes, fasting glucose, and glycosylated hemoglobin A1c (HbA1c). These data were obtained from the hospital medical records.

### Measurement of Platelet Function

The platelet function was assessed using a thromboelastography (TEG) Analyzer 5000 (Haemonetics Corporation, USA) with assays taken within 1 h following blood sample collection. Peripheral venous whole blood was collected by venipuncture in vacutainer tubes containing 3.2% sodium citrate and sodium heparin (Becton–Dickinson, San Jose, CA) at least 7 days after patients had received DAPT and 12 h after the last dose in our study. The TEG was performed using standard methods in hospital laboratories according to manufacturers' instructions. Four channels were used to detect the effects of antiplatelet therapy with arachidonic acid (AA) and adenosine diphosphate (ADP) activators at 37°C. The maximum amplitude (MA) was designated as the maximum intensity obtained from a clot, which represented the maximum platelet function that observed in a blood sample. MA_ADP_ represented the ADP-induced clot strength, MA_AA_ was the AA-induced clot strength, MA_fibrin_ was the activator-induced clot strength (measurement of fibrin contribution), and MA_thrombin_ was the thrombin-induced clot strength. The platelet inhibition rate induced by AA or ADP was calculated by computer software according to the following formula: Inhibition rate (%) = [(MA_thrombin_ – MA_ADP_ or MA_AA_)/(MA_thrombin_ – MA_fibrin_)] × 100%, which represented the responsiveness to aspirin or clopidogrel. AA% < 50% can be considered as aspirin HRPR and ADP% < 30% or MA_ADP_ > 47 mm can be considered as clopidogrel HRPR (21, 22).

### Statistical Analysis

Statistical analysis was performed using IBM SPSS 22.0 software (IBM Corp., Armonk, NY, USA). Categorical variables were presented as percentages and frequencies, whereas continuous data were presented as the mean ± the standard deviation (SD), or median [interquartile range] for data having a skewed distribution. To detect deviations from a normal distribution the Kolmogorov–Smirnov test was used. Comparisons between groups were evaluated using two-sided *t*-tests, the Mann–Whitney U test, or the Chi-square test according to the respective distribution. A *P*-value < 0.05 was considered statistically significant.

We classified all patients into DM and non-DM groups. Propensity score matching (PSM) analysis applied logistic regression to balance the distribution of possible confounding variables between groups, including age, hypertension, dyslipidemia, and coronary heart disease. Patients were matched between the groups using the nearest logit of the propensity score and a PSM ratio of 1:1. For further analysis, patients were classified into HRPR and non-HRPR groups with respect to clopidogrel responsiveness. Different variables between the two groups (*P* < 0.05) were conducted multivariate logistic regression analysis to identify independent risk factors of clopidogrel HRPR. The results were expressed as odd ratios (ORs) with 95% confidence intervals (95% CI).

## Results

### Demographics and Clinical Characteristics

**Figure 1:**
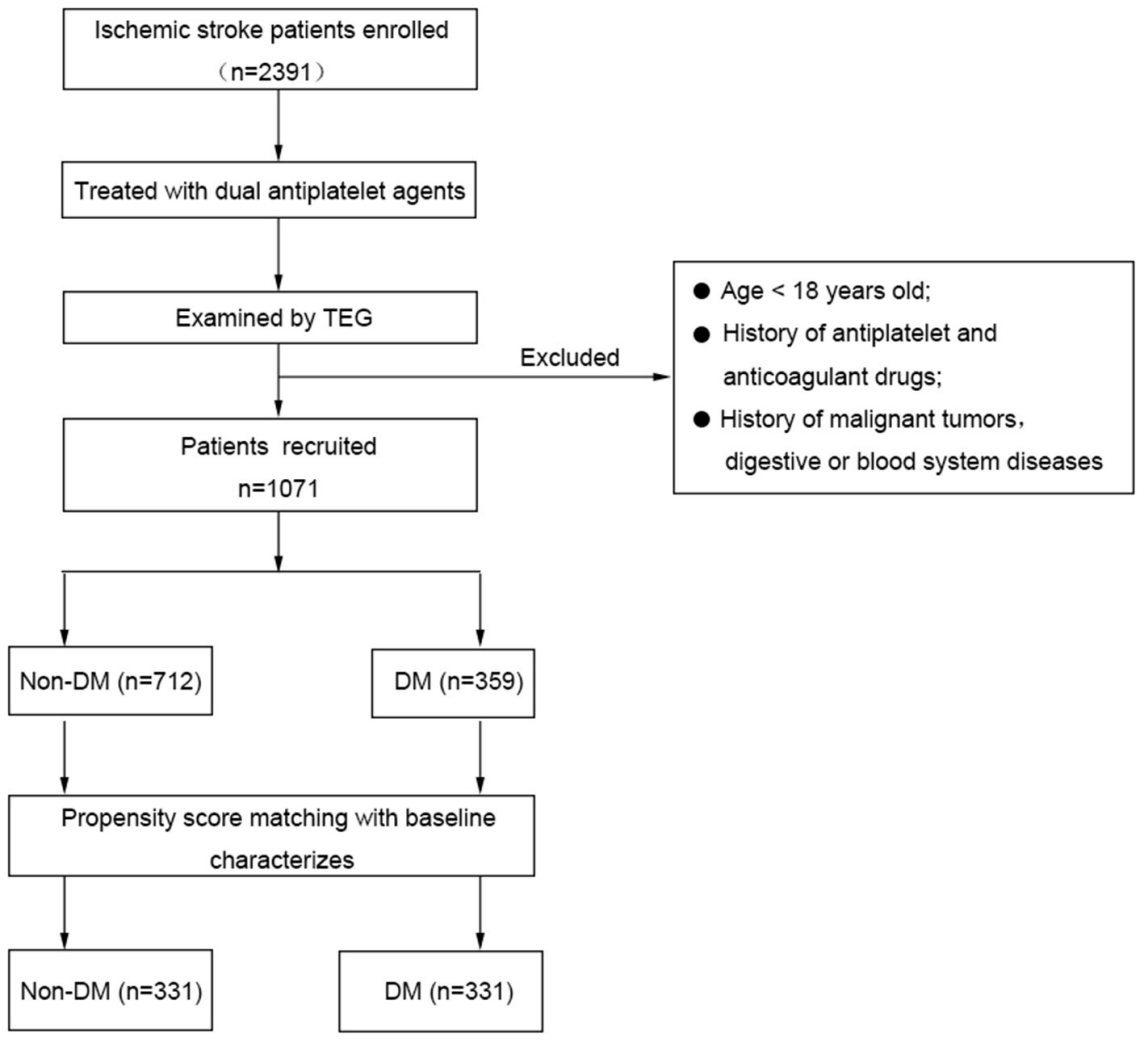
Flowchart of patient recruitment.

We have included 1,071 patients with acute ischemic stroke in this study (Figure 1). The demographic and clinical data of DM group (*n* = 712) and non-DM group (*n* = 359) are presented in Table 1. No statistical differences were found in sex, cerebrovascular risk factors (smoking, alcohol intake, history of stroke/TIA), and platelet indices between the two groups. In addition, age, prevalence of cerebrovascular risk factors (hypertension, hyperlipidemia, coronary heart disease), creatinine, fasting glucose and HbA1c were significantly different between the DM and non-DM group (*P* < 0.05).

**Table 1:** Patients' baseline characteristics before and after PS matching.

**Characteristics**	**Before PS matching**			**After PS matching**		
	**Non-DM (*n* = 712)**	**DM (*n* = 359)**	***P*-value**	**Non-DM (*n* = 331)**	**DM (*n* = 331)**	***P*-value**
Age (*y*)	56.0 [50.0–65.0]	60.0 [53.0–65.0]	**<0.001**	59.0 [53.0–65.0]	60.0 [53.0–65.0]	0.848
Male, *n* (%)	525 (73.7)	253 (70.5)	0.258	238 (71.9)	233 (70.4)	0.668
Smoking, *n* (%)	362 (50.8)	162 (45.1)	0.077	170 (51.4)	151 (45.6)	0.140
Alcohol intake, *n* (%)	302 (42.4)	140 (39.0)	0.283	145 (43.8)	129 (39.0)	0.207
**Medical history**, ***n*** **(%)**						
History of stroke/TIA	130 (18.3)	74 (20.6)	0.354	66 (19.9)	64 (19.3)	0.845
Hypertension	442 (62.1)	274 (76.3)	**<0.001**	253 (76.4)	253 (76.4)	1.000
Hyperlipidemia	58 (8.1)	58 (16.2)	**<0.001**	35 (10.6)	35 (10.6)	1.000
Coronary heart disease	48 (6.7)	46 (12.8)	**<0.001**	33 (10.0)	33 (10.0)	1.000
**Laboratory data**						
Creatinine (umol/L)	73.0 [64.0–85.0]	70.0 [59.0–83.0]	**0.008**	74.0 [64.0–86.0]	70.0 [59.0–83.0]	**0.004**
eGFR (ml/min/1.73 m^2^)	94.6 [82.5–103.8]	94.6 [82.4–103.3]	0.719	91.0 [80.0–101.0]	95.0 [83.0–103.0]	0.049
Platelet count (*10^9^/L)	213 [178–251]	210 [176–256]	0.530	212 [176–246]	213 [178–259]	0.377
PDW (fL)	13.1 [11.7–15.0]	13.3 [11.9–15.2]	0.197	13.0 [12.0–15.0]	13.0 [12.0–15.0]	0.235
MPV (L)	10.9 [10.1–11.7]	11.0 [10.3–11.8]	0.221	11.0 [10.0–12.0]	11.0 [10.0–12.0]	0.161
P-LCR (%)	32.2 [27.0–39.2]	33.2 [27.5–39.7]	0.213	32.0 [27.0–39.0]	33.0 [27.0–40.0]	0.162
Fasting glucose (mmol/L)	5.1 [4.8–5.5]	6.9 [5.6–8.8]	**<0.001**	5.0 [5.0–6.0]	7.0 [6.0–9.0]	**<0.001**
HbA1c (%)	5.6 [5.5–5.8]	7.3 [6.5–8.6]	**<0.001**	6.0 [5.0–6.0]	7.0 [7.0–9.0]	**<0.001**

*Data given as n (%) or median [interquartile range]. PS, propensity score; DM, Diabetes mellitus; TIA, transient ischemic attack; eGFR, glomerular filtration rate; PDW, platelet distribution width; MPV, mean platelet volume; P-LCR, platelet large cell ratio; HbAc1, glycosylated hemoglobin A1c. The bold values indicates P-value < 0.05*.

To reduce differences of baseline characteristics, the PSM was further analyzed to balance the distribution of variables between the groups (Table 1). After PSM, the total number of patients in each study group was 331. There were no significant differences in baseline characteristics between the two groups, except for creatinine, fasting glucose and HbA1c.

### The Impact of DM on Platelet Function

Before PSM, we found that the median of MA (63.0 vs. 62.0 mm, *P* < 0.05) and MA_ADP_ (34.6 vs. 30.3 mm, *P* < 0.05) was significantly higher in the DM group and the ADP% (55.7% vs. 60.3%, *P* < 0.05) was significantly lower in the DM group compared with the non-DM group. No significant differences in the median levels of AA% were observed between the two groups (96.0% vs. 97.0%, *P* > 0.05).

After PSM, the difference in the median levels of MA (63.1 vs. 62.5 mm, *P* < 0.05), MA_ADP_ (34.6 vs. 29.3 mm, *P* < 0.05) and ADP% (55.8 vs. 67.4%, *P* < 0.05) between the DM and non-DM group (Table 2) remained. However, the median levels of AA% were significantly higher in the DM-group than those without (96.1% vs. 93.0%, *P* < 0.05).

**Table 2:** Platelet reactivity, high residual on-treatment platelet reactivity (HRPR) before and after PS matching.

**Variables**	**Before PS matching**			**After PS matching**		
	**Non-DM (*n* = 712)**	**DM (*n* = 359)**	***P*-value**	**Non-DM (*n* = 331)**	**DM (*n* = 331)**	***P*-value**
MA (mm)	62.0 [58.1–65.6]	63.0 [59.8–66.3]	**0.002**	62.5 [58.5–65.5]	63.1 [59.9–63.1]	**0.032**
MA_ADP_ (mm)	30.3 [16.9–41.0]	34.6 [22.5–44.9]	**0.001**	29.3 [14.7–40.4]	34.6 [22.6–45.0]	**<0.001**
ADP% (%)	60.3 [39.9–84.3]	55.7 [36.7–80.1]	**0.039**	67.4 [42.2–87.1]	55.8 [36.7–80.2]	**0.004**
AA% (%)	97.0 [82.8–100]	96.0 [80.0–100]	0.306	93.0 [69.8–99.5]	96.1 [78.0–100.0]	**0.011**
Clopidogrel HRPR, *n* (%)	123 (17.3)	81 (22.6)	**0.038**	52 (15.7)	76 (23.0)	**0.018**
Aspirin HRPR, *n* (%)	89 (12.6)	43 (12.2)	0.818	51 (15.4)	41 (12.4)	0.268

*Data given as n (%) or median [interquartile range]. PS, propensity score; DM, Diabetes mellitus; MA, maximum amplitude; MA_ADP_, ADP-induced platelet-fibrin clot maximum amplitude; ADP%, inhibition rate of adenosine diphosphate (ADP); AA%, inhibition rate of arachidonic acid (AA); HRPR: high residual on-treatment platelet reactivity. The bold values indicates P-value < 0.05*.

Among all study populations 19.0% of patients exhibited clopidogrel HRPR. The prevalence rate of clopidogrel HRPR was significantly lower in the non-DM group compared with the DM group (17.3% vs. 22.6%, *P* < 0.05), but no difference in aspirin HRPR was found between the two groups (Table 2). The results were the same before and after PSM.

### Risk Factors of Clopidogrel HRPR

Previous studies have demonstrated that clopidogrel HRPR is affected by many factors. Accordingly, we classified the patients into two groups (non-HRPR and HRPR). The demographic and clinical data of the two groups were compared. Age, sex, smoking, alcohol intake, DM, creatinine and HbA1c were significantly different between the groups (Table 3). After adjustment for potential confounders (age, sex, smoking, alcohol intake, creatinine and HbA1c), the DM (OR = 1.48, 95% CI: 1.03–2.07, *P* < 0.05) remained independent of the clopidogrel HRPR.

**Table 3:** Risk factors for clopidogrel high residual on-treatment platelet reactivity (clopidogrel HRPR) in all patients.

**Characteristics**	**All patients**		
	**Non-HRPR** **(*n* = 867)**	**HRPR** **(*n* = 204)**	***P*-value**
Age (*y*)	57.0 [50.0–65.0]	60.0 [53.0–66.0]	**<0.001**
Male, *n* (%)	661 (76.2)	117 (57.4)	**<0.001**
Smoking, *n* (%)	449 (51.8)	75 (36.8)	**<0.001**
Alcohol intake, *n* (%)	374 (43.1)	68 (33.3)	**0.001**
**Medical history**, ***n*** **(%)**			
History of stroke/TIA	158 (18.2)	46 (22.5)	0.157
Hypertension	577 (66.6)	139 (68.1)	0.665
Diabetes mellitus	278 (32.1)	81 (39.7)	**0.038**
Hyperlipidemia	88 (10.1)	28 (13.7)	0.139
Coronary heart disease	70 (8.1)	24 (11.8)	0.094
**Laboratory data**			
Creatinine (umol/L)	73.0 [63.0–84.3]	70.0 [57.0–84.0]	**0.008**
eGFR (ml/min/1.73 m^2^)	94.8 [83.0–104.4]	94.3 [81.3–102.4]	0.719
Platelet count (*10^9^/L)	213 [177–253]	210 [177–250]	0.530
PDW (fL)	13.2 [11.8–13.2]	13.2 [11.9–14.8]	0.197
MPV (L)	10.9 [10.2–11.7]	11.0 [10.3–11.7]	0.221
P-LCR (%)	32.5 [27.8–39.6]	33.0 [27.7–38.7]	0.213
Fasting glucose (mmol/L)	5.3 [4.8–6.3]	5.4 [5.0–6.6]	0.853
HbA1c (%)	5.8 [5.5–6.5]	5.9 [5.6–6.9]	**<0.001**

*Data given as n (%) or median [interquartile range]. HRPR: high residual on-treatment platelet reactivity; TIA, transient ischemic attack; eGFR, glomerular filtration rate; PDW, platelet distribution width; MPV, mean platelet volume; P-LCR, platelet large cell ratio; HbAc1, glycosylated hemoglobin A1c. The bold values indicates P-value < 0.05*.

## Discussion

In this study, we found that patients with DM had higher levels of MA, MA_ADP_ and lower levels of ADP%. The clopidogrel HRPR rate was also higher in DM group than non-DM group. Additionally, after adjustment for potential confounders, DM remained an independent risk factor of HRPR following clopidogrel therapy. These results suggested that in acute ischemic stroke patients taking DAPT, DM is associated not only with an increase of platelet reactivity but also with a low responsiveness to clopidogrel.

Although it is widely recognized that DM could influence the platelet reactivity in patients following percutaneous transluminal coronary intervention (23, 24), the data examining the DM influences on platelet function in acute ischemic stroke patients taking DAPT have been sparse. Previous studies have found that in ischemic stroke patients receiving clopidogrel (75 mg) daily for 1 week, clopidogrel resistance is associated with DM (25). In a study involving 237 patients following recent ischemic stroke or TIA treated receiving DAPT, DM was found to be associated with HRPR defined as aspirin resistance or clopidogrel resistance (26), which was different from our study. Our study provides further evidence that DM is associated with clopidogrel HRPR but not with aspirin HRPR.

In our study, AA-mediated platelet reactivity and aspirin HRPR were not affected by DM. The relationship between DM and aspirin HRPR remained controversies. As reported by previous studies (27), despite a high risk of aspirin HRPR in DM patients, DM itself does not contribute to a higher risk of aspirin HRPR and is more likely to be related to insulin resistance. In another study, it is found that aspirin resistance was more common among participants treated with low dose aspirin compared with higher doses (28). We speculate that different methods and time of platelet function tested, differences in aspirin dosing and frequency, and characteristics of the patient populations studied would affect the results of platelet function test. In future studies, we will try to explore which factors have effects on the relationship between DM and aspirin HRPR.

Studies have shown that clopidogrel HRPR is 4–30% in patients who use conventional doses of clopidogrel (29) and rises to 28% in ischemic cerebrovascular disease (30–32). Moreover, up to 40% of patients exhibiting clopidogrel HRPR may have recurring thrombotic events (33, 34). Therefore, the prediction and identification of patients with HRPR is a significant issue, as they may benefit from other antiplatelet drugs for the prevention of ischemic events and improvement of clinical outcomes. In our study, ADP-mediated platelet reactivity and clopidogrel HRPR was associated with DM. Therefore, the presence of DM should be considered in developing strategies for anti-platelet treatment of acute ischemic stroke patients.

The mechanism underlying increased platelet reactivity and clopidogrel HRPR in DM remains unclear. One possible reason is that chronic inflammatory reaction accompanies DM, which can upregulate the expression of cyclooxygenase-2 (COX-2) in vascular endothelial cells, monocyte-macrophages and other cells, leading to a significant increase in platelet activity (35, 36). In addition, DM patients have a high level of glycosylation of platelet surface membrane proteins, which competitively inhibits their acetylation and weakens the anti-platelet aggregation effect of clopidogrel (37).

Some limitations should be addressed. First, in this retrospective study, although most of the measured confounders were balanced between the two groups by PSM, bias may still exist. Confounding factors such as the use of proton pump inhibitors and the types of use, the use of statin, liver function or heterogeneity of metabolic genes (e.g., CYP2C19 gene type) may affect the responsiveness of clopidogrel drugs. However, we have included as many confounding factors as possible. Second, the degree of platelet aggregation and its response also depends on the type of diabetes. Type 2 diabetes has a higher ADP-induced aggregation rate than type 1, although due to the current sparsity of data conclusions are inconsistent. Third, our research subjects are Chinese patients, so generalizing these results to non-Asian patients may need careful interpretation and further research.

In conclusion, we found that in acute ischemic stroke patients taking DAPT, DM was associated with increased platelet activity and a greater prevalence of clopidogrel HRPR. Hence, it is necessary for us to stratify patients according to the presence or absence of DM and to choose a personalized treatment strategy to reduce the risk of ischemic events.

## Data Availability Statement

The raw data supporting the conclusions of this article will be made available by the authors, without undue reservation.

## Ethics Statement

The studies involving human participants were reviewed and approved by the Ethics Board of Tongji Hospital. Written informed consent for participation was not required for this study in accordance with the national legislation and the institutional requirements.

## Author Contributions

YG, YZ, and LW collected the clinical data. YG, DH, and JZ processed statistical data. YG, ZY, and HH drafted and revised the manuscript. XL and HH designed and guided the study. All authors contributed to the article and approved the submitted version.

## Funding

This study was supported by the National Nature Science Foundation of China (81771341 to XL), the Flagship Program of Tongji Hospital (2019CR106 to XL), the second batch of clinician research projects of HUST to XL, and the Natural Science Foundation of Guangdong Province (2018A030313820 to DH). Guangdong Provincial Engineering Center for Major Neurological Disease Treatment, Guangdong Provincial Translational Medicine Innovation Platform for Diagnosis and Treatment of Major Neurological Disease, Guangdong Provincial Clinical Research Center for Neurological Diseases.

## Conflict of Interest

The authors declare that the research was conducted in the absence of any commercial or financial relationships that could be construed as a potential conflict of interest.

## Publisher's Note

All claims expressed in this article are solely those of the authors and do not necessarily represent those of their affiliated organizations, or those of the publisher, the editors and the reviewers. Any product that may be evaluated in this article, or claim that may be made by its manufacturer, is not guaranteed or endorsed by the publisher.
