# Supplementary material for: Impact of Diabetes on Platelet Function in Acute Ischemic Stroke Patients Taking Dual Antiplatelet Therapy
Source: Front Neurol. 2021 Nov 4;12:712024. doi: 10.3389/fneur.2021.712024 (PMC8599121; doi:10.3389/fneur.2021.712024)
Supplement: Supplementary file 1 [file Table_1.docx]

# Supplemental Data

**Table S1. Risk factors for clopidogrel high residual on-treatment platelet reactivity (clopidogrel HRPR) in all patients.**

| **Characteristics** | **All patients** | | |
| --- | --- | --- | --- |
|  | **Non-HRPR (n=867)** | **HRPR (n=204)** | ***P* Value** |
| Age (y) | 57.0 [50.0–65.0] | 60.0 [53.0–66.0] | **＜0.001** |
| Male, n (%) | 661 (76.2) | 117 (57.4) | **＜0.001** |
| Smoking, n (%) | 449 (51.8) | 75 (36.8) | **＜0.001** |
| Alcohol intake, n (%) | 374 (43.1) | 68 (33.3) | **0.001** |
| **Medical history, n (%)** |  |  |  |
| History of stroke/TIA | 158 (18.2) | 46 (22.5) | 0.157 |
| Hypertension | 577 (66.6) | 139 (68.1) | 0.665 |
| Diabetes mellitus | 278 (32.1) | 81 (39.7) | **0.038** |
| Hyperlipidemia | 88 (10.1) | 28 (13.7) | 0.139 |
| Coronary heart disease | 70 (8.1) | 24 (11.8) | 0.094 |
| **Laboratory data** |  |  |  |
| Creatinine (umol/L) | 73.0 [63.0–84.3] | 70.0 [57.0–84.0] | **0.008** |
| eGFR (ml/min/1.73m2) | 94.8 [83.0–104.4] | 94.3 [81.3–102.4] | 0.719 |
| platelet count (*10^9^/L) | 213 [177–253] | 210 [177–250] | 0.530 |
| PDW (fL) | 13.2 [11.8–13.2] | 13.2 [11.9–14.8] | 0.197 |
| MPV (L) | 10.9 [10.2–11.7] | 11.0 [10.3–11.7] | 0.221 |
| P-LCR (%) | 32.5 [27.8–39.6] | 33.0 [27.7–38.7] | 0.213 |
| Fasting Glucose (mmol/L) | 5.3 [4.8–6.3] | 5.4 [5.0–6.6] | 0.853 |
| HbA1c (%) | 5.8 [5.5–6.5] | 5.9 [5.6–6.9] | **＜0.001** |

Data given as n (%) or median [interquartile range]; Abbreviations: HRPR: high residual on-treatment platelet reactivity; TIA, transient ischemic attack; eGFR, glomerular filtration rate; PDW, platelet distribution width; MPV, mean platelet volume; P-LCR, platelet large cell ratio; HbAc1, glycosylated hemoglobin A1c
